# Cholera outbreak in Addis Ababa, Ethiopia: A case-control study

**DOI:** 10.1371/journal.pone.0235440

**Published:** 2020-07-02

**Authors:** Getachew Dinede, Abdulnasir Abagero, Tadele Tolosa

**Affiliations:** 1 Epidemiology Directorate, Ministry of Agriculture, Addis Ababa, Ethiopia; 2 Field Epidemiology Training Program, School of Public Health, Addis Ababa University, Ethiopia; 3 Microbiology and Dairy Heard Health Management, School of Veterinary Medicine, College of Agriculture and Veterinary Medicine, Jimma University, Jimma, Ethiopia; Rabin Medical Center, Beilinson Hospital, ISRAEL

## Abstract

**Background:**

Cholera remains a significant public health problem in more than one-third of the countries of the world. Cholera outbreak has become more common in Addis Ababa particularly in the rainy seasons; however, there is a paucity of data on risk factors associated with cholera outbreaks rendering interventions difficult. We investigated the outbreak to identify its etiology, source, risk factors and in order to control the outbreak.

**Methods:**

We compared cases with health center-based unmatched controls (1:2). Cases were patients aged ≥5 years with acute watery diarrhea, with or without vomiting while controls were persons aged ≥5 years without history of acute watery diarrhea. We interviewed our study participants using structured questionnaire to collect demographic and cholera risk factors data. We described the outbreak over time, and then tested our hypotheses using unconditional logistic regression.

**Results:**

The outbreak began on 7 September, 2017 reaching its peak on 23 September, 2017 and ended on 01 October, 2017. We identified a total of 25 cases (Median age: 38 years; IQR: 20 years) and recruited 50 controls (Median age: 35 years; IQR: 29 years). All case-patients had acute watery diarrhea and dehydration requiring intravenous fluids. All cases were admitted to cholera treatment center but there were no deaths. Stool and water samples yielded isolates of *Vibrio cholerae* O1 of serological subtype Ogawa. Consumption of contaminated holy water (AOR: 20.5, 95%CI: 3.50, 119.61) and raw vegetables (AOR: 15.3, 95%CI: 3, 81.51) were independent risk factors whereas washing hands with soap after visiting latrine (AOR: 0.04, 95%CI: 0.01, 0.25) was independent protective factor.

**Conclusion:**

Our findings demonstrated cholera foodborne transmission via consumption of raw vegetables, and its waterborne transmission via consumption of contaminated holy water. Washing hands with soap after visiting latrine was protective. We recommended cooking of vegetables and promoting hand washing.

## Background

Cholera is an acute diarrheal illness caused by infection of intestine with *Vibrio cholerae*, of usually serogroup O1. It is primarily waterborne illness, but it can also be transmitted via contaminated food and, rarely, between infected persons. Cholera has an incubation period that can range from several hours to 5 days. Onset of illness may be sudden, with profuse, watery diarrhea, or there can be premonitory symptoms such as anorexia, abdominal discomfort, and simple diarrhea. Vomiting is often present, occurring a few hours after the onset of diarrhea [1–3].

The true global burden of cholera is largely unknown as the passively-acquired surveillance data underreports its cases related to poor surveillance systems and laboratory diagnostic capacities, and social, political and economic disincentives for reporting in many cholera-endemic countries. In consistent with this finding, the World Health Organization estimates that only 5–10% of the cholera cases occurring annually are officially reported by Member countries [4]. Ali, *et al*. [5] estimated that there have been approximately 1.3 billion people who are at risk for cholera in endemic countries worldwide, with an estimated 2.86 million cases and 95,000 deaths of cholera occurring annually, with Sub-Saharan Africa accounting for 60% of the estimated cases. The study also estimated in Ethiopia that nearly 70 million people are at risk of cholera with an estimated 275,221 cases and 10, 458 deaths occurring annually, representing incidence rate of 4 cases per 1000 populations.

Understanding of risk factors associated with cholera outbreaks aids to identify practices that expose the community to cholera infection, which in turn permits to design plausible behavior change interventions. Previous studies have identified the most known risk factors for cholera outbreak in different parts of the world including contaminated water sources, unwashed raw vegetables, lack of adequate latrines and, heavy rains and floods [6]. Although representative studies have not been conducted in Ethiopia to identify risk factors associated with cholera outbreak nationwide, limited studies have been conducted identifying different cholera outbreak risk factors including unsanitary latrine, contact with cholera cases and travel history to cholera outbreak areas [7, 8] and, poor sanitation and insufficient access to clean water [9]. Cholera outbreak has become more common in Addis Ababa particularly in the rainy seasons; however, there is a paucity of data on risk factors associated with these cholera outbreaks rendering interventions difficult. Therefore, this study was aimed to investigate the outbreak to identify its etiology, source, risk factors, and in order to control the outbreak.

## Methods

### Study area

Addis Ababa, the capital of Ethiopia, is the industrial, commercial and cultural center of the country. Administratively, Addis Ababa is divided into ten sub-cities and sub-cities are further divided into 118 woredas (also called districts). Addis Ababa has a total population of 3,352,000 accounting for 4.3% and 40% of the population of the country and urban, respectively [10] having population density of 165.1/km^2^ and total land area of 540km^2^ [11].

In Addis Ababa, in 2016, there were a total of 92 public health centers and 11 public hospitals, with health centers to population ratio of 1:36435 and hospitals to population ratio of 1:304727. In the same year, there were 8765 health professionals in Addis Ababa, with 283(3.23%) of them being general practioners medical doctors and 126(1.44%) specialist medical doctors representing population ratio of 8.4 and 3.8 per 100000 populations, respectively [12].

### Study design

We described the outbreak in person and over time using descriptive epidemiology to generate hypotheses about possible exposures that were common to the cases. Then, we conducted health center-based unmatched case-control study (between 07 September, 2017 and 01 October, 2017).

### Definitions

We defined the study population as a person aged 5 years or more living in Addis Ababa between 07 September and 01 October 2017. A suspected case of cholera was defined as a patient aged 5 years or more with acute watery diarrhea, with or without vomiting during the outbreak (between 07 September and 01 October 2017). Whereas, a case of cholera was defined as confirmed when *Vibrio cholerae* is isolated from any suspected case-patient of cholera. [13]. We also defined controls as patients aged 5 years or more without history of acute watery diarrhea. We defined the referent exposure duration as 5 days prior to clinical signs and symptoms (for case-patients) or prior to selection (for controls). In this article, holy water refers to waters blessed by priests and used for religious ceremonies in Orthodox Christian churches.

### Cases and controls selection

We searched for suspected cases of cholera in health centers. In Addis Ababa, health centers serve a defined administrative area called districts, with all districts having at least one health center. To improve ascertainment of cases, health extension workers and surveillance officers were initiated to conduct awareness creation on the symptoms of cholera to the community so that individuals with signs of cholera would immediately visit health centers in their vicinity. All cases fulfilling the suspected case definition were recruited. Suspected cases of cholera who were identified at health centers were transferred to cholera treatment center for further case management. Upon detection of cases, we selected two unmatched controls for each case (2:1) from the same health center where we recruited the suspected case of cholera.

### Environmental investigation

Preliminary analysis of the outbreak data demonstrated that nearly half (48%) of the cases drank holy water from two holy water sources. We selected these holy water sites which were located in the compounds of two Orthodox Christian churches to investigate their hygienic status using semi-structured questionnaires. These holy waters are surface waters, which are small pools. Believers either drink this holy water during the religious ceremony at the church or take home in any container for later use. In contrast to regular drinking water, holy water is consumed in a very small amount as it has been believed that holy water has spiritual power to heal illnesses and to cast out demons. We also selected the houses of five cases who drank holy water purposively to assess their drinking water, latrine and environmental conditions using semi-structured questionnaires.

### Data collection

We line listed the cases and reviewed their medical records to collect information on signs and symptoms, and laboratory results. In addition, we interviewed each of the suspected cases and controls using structured questionnaire. We were unable to pre-test our questionnaire because of financial and time constraints. However, we designed our questionnaire by reviewing standard cholera outbreak investigation forms to tailor to our study based on the information needed to collect. The questionnaire was written in English but the interviewer translated into the local language (Amharic) orally while interviewing the study participants. Data on demographic characteristics and different potential risk factors including drinking water (source, treatment), feeding habits (unwashed fruits, raw vegetables, raw meat, eating food outside home), availability of latrine, history of contact with suspected cases of cholera and travel history were collected using the questionnaire. We also investigated case-patients’ houses to collect information on their drinking water (source, storage, treatment), and latrine (type, shared/unshared, availability of soaps). Furthermore, we investigated two holy water sites using checklists to assess their hygienic status.

### Sample collection and processing

Stool samples were collected from only the first seven cholera case-patients and transported to the laboratory in Cary-Blair media. In addition, water samples were collected from two holy water sources in 500mL bottles. Both the stool and water samples were analyzed at Addis Ababa Health Bureau Public Health Research and Emergency Management Laboratory.

A 0.22μl filter papers were used to filter water samples, and the membrane filters were then enriched in alkaline peptone water (pH 8.4) at 37°C for 4–6h, and then cultured on selective media, as described previously [14]. Stool samples were also subjected to alkaline peptone water enrichment and then cultured in the same procedure as the water samples.

### Isolation and identification of *V*.*cholerae*

Following culturing on the selective media, typical *V*.*cholerae* colonies were isolated and identified as described previously [15]. While polyvalent antisera slide agglutination test was employed to identify *V*.*cholerae* serogroups, serotype-specific monovalent Inaba and Ogawa antisera were used to identify serological subtypes of *V*.*cholerae* [14].

### Data analysis

Data was cleaned and entered into IBM SPSS (Version 20.0. Armonk, NY: IBM Corp.). We described the outbreak in terms of time. We assessed demographic characteristics distribution in cases and controls using Pearson Chi-square. But we used Fischer exact test when expected frequencies in the cells were less than 5. Logistic regression model fitness was tested using Cox and Snell R square, with >0 being the best fit. We compared cases with controls in terms of their exposure behavior to the risk factors using bivariate (Crude odds ratio [COR]) and multivariate (adjusted odds ratio [AOR]) logistic regression analysis. We used forward stepwise selection method to select variables that were kept in the final multivariable model. We used 95% confidence level and less than 5% level of significance (p<0.05) to test our hypotheses.

### Ethics statement

Research Ethical Review Committee of Addis Ababa Health Bureau approved the study. The national public health authorities are empowered and mandated to conduct outbreak investigation as indicated in the Ethiopian Public Health Institute Establishment Council of Ministers Regulation No.301/2013 [16] to protect the community from outbreak adverse effects compelling the community to participate during outbreak investigations as they are benefitted from the interventions. We obtained oral consent from our study participants and protected their individual privacy using confidential codes, with analysis of the outbreak data anonymously.

## Results

### Descriptive epidemiology

**Fig 1:**
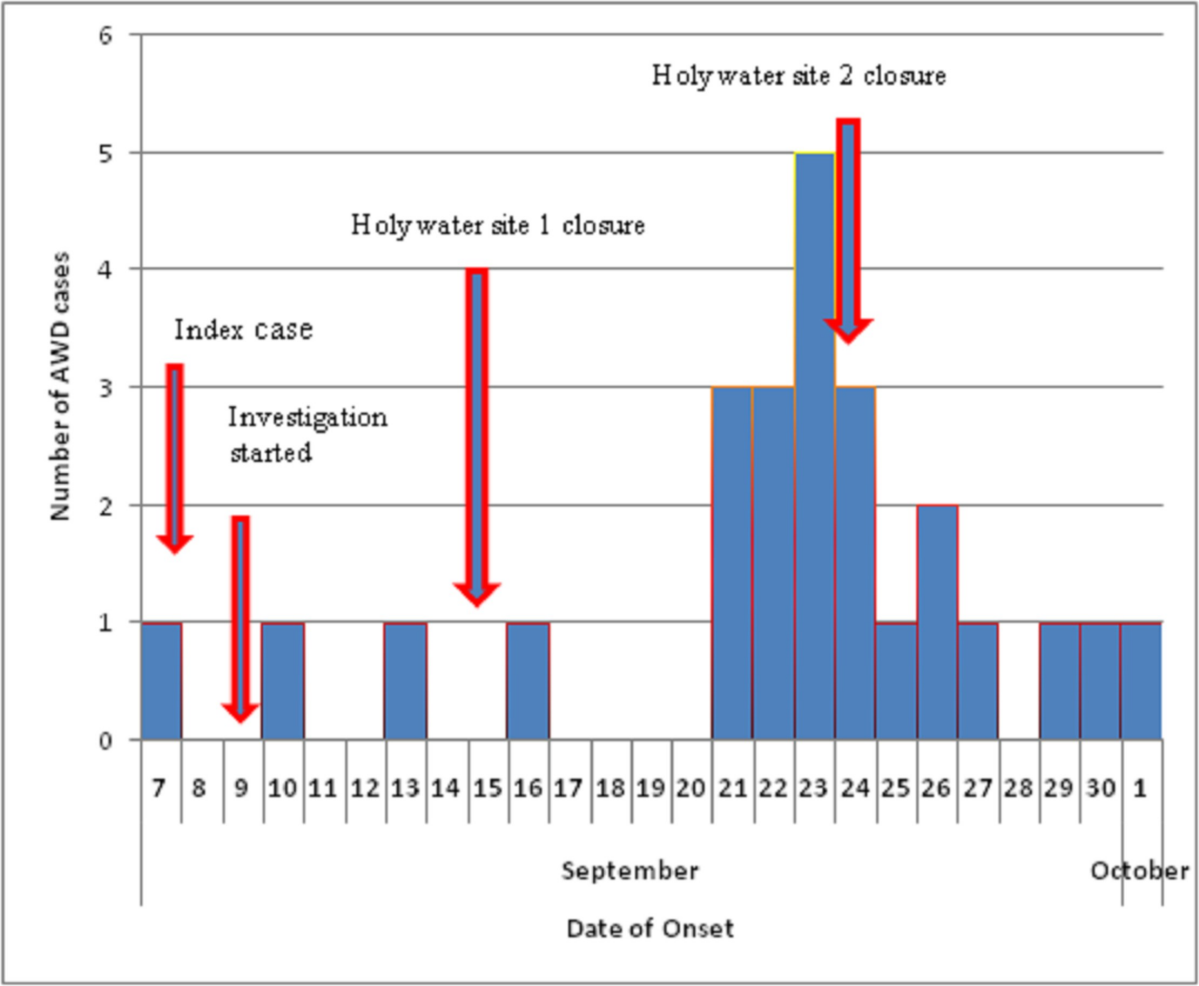
Cholera cases by date of onset, Addis Ababa, Ethiopia: 07 September to 01 October, 2017.

The outbreak began on 07 September 2017 reaching its peak at 23 September and ended at the beginning of October, 2017 (Fig 1). A 46 year-old index case was notified to Addis Ababa Health Bureau on 08 September 2017. He was admitted to Zewditu Hospital Cholera Treatment Center on 08 September 2017. He drank contaminated holy water on 01 September 2017 but he did not have history of contact with cholera suspected person and of travel to cholera affected areas five days before his onset of illness. He experienced acute watery diarrhea, vomiting and dehydration, with isolation of *Vibrio cholerae* from his stool sample. In addition, the water sample collected from where he drank holy water was found to be *Vibrio cholerae* positive. He was discharged on 12 September 2017 following his recovery.

During this outbreak, 25 case-patients were identified (median age: 38 years; IQR: 20 years), and all of them were included in our study. Out of these, seven cases were laboratory confirmed while the rest were classified as cholera cases based on case definition. All of the cases were admitted to cholera treatment center but there were no deaths. All case-patients had acute watery diarrhea and dehydration requiring intravenous fluids, with some case-patients experiencing vomiting as well.

Higher percentages of controls were with no formal education than cases, however, our univariate analysis did not show statistical significance. We found that higher percentages of cases were unemployed than controls but our analysis did not indicate statistical significance. Furthermore, the finding showed that cases and controls had almost similar distribution with regard to sex, age and marital status in their respective categories (Table 1). Our laboratory isolation and identification procedures for *Vibrio cholerae* demonstrated that all of the stool specimens from seven case-patients, and the water specimens collected from holy water sources yielded isolates of *Vibrio cholerae* O1 of serological subtype Ogawa.

**Table 1:** Demographic characteristics of cholera cases and controls (univariate analysis), Addis Ababa, Ethiopia: 07 September to 01 October, 2017.

Socio-demographic characteristics	Cases(n = 25)	Controls(n = 50)	p-value
	**Number (%)**	**Number (%)**	
**Sex**			
Male	15(60.00)	28(56.00)	0.74
Female	10(40.00)	22(44.00)	
**Age(years)**			
15–24	3(12.00)	9(18.00)	0.46
25–34	7(28.00)	15(30.00)	
35–44	3(12.00)	11(22.00)	
44+	12(48.00)	15(30.00)	
**Marital status**			
Single	9(36.00)	20(40.00)	0.57
Married	12(48.00)	26(52.00)	
Widowed	4(16.00)	3(6.00)	
Divorced	0(0.00)	1(2.00)	
**Education**			
None	3(12.00)	12(24.00)	0.24
Primary(grades 1–8)	8(32.00)	14(28.00)	
Secondary(grades 9–12)	9(36.00)	9(18.00)	
Tertiary	5(20.00)	15(30.00)	
**Occupation**			
Government employee	4(16.00)	15(30.00)	0.34
Private employee	7(28.00)	17(34.00)	
Self-employed	1(4.00)	1(2.00)	
Unemployed	13(52.00)	17(34.00)	

### Case-control study

We enrolled 25 cases (Median age: 38 years; IQR: 20 years) and 50 health center-based controls (Median age: 35 years; IQR: 29 years) for the case-control study. In our bivariate analysis, we did not find statistically significant association of sex, age, marital status, education and occupation with cholera illness (Table 2). Regarding cholera exposures, consumption of raw vegetables and drinking contaminated holy water, and contact history with cholera case were statistically associated risk factors for cholera whereas washing hands with soap was significant protective factor against cholera. However, we did not find statistically significant association of consumption of raw fruits, raw meat, food outside home and water from shared water sources, treating drinking water with chemicals, and travel history with cholera illness (Table 3).

**Table 2:** Comparing demographic characteristics among cholera cases and controls using bivariate analysis, Addis Ababa, Ethiopia: 07 September to 01 October, 2017.

Demographic characteristics	Cases Number (%)	Controls Number (%)	COR at (95%CI)^¶^
**Sex**			
Male	15(60)	28(56)	1.18(0.44,3.13)
Female	10(40)	22(44)	
**Age**			
15–34	10(40)	24(48)	0.72(0.27–1.91)
≥35	15(60)	26(52)	
**Marital status**			
Married	12(48)	26(52)	0.85(0.32–2.23)
Unmarried (single, divorce, widowed)	13(52)	24(48)	
**Education**			
None/primary	11(44)	26(52)	0.73(0.28–1.90)
Secondary/tertiary	14(56)	24(48)	
**Occupation**			
Employed (government, private, self)	12(48)	33(66)	0.48(0.18–1.27)
unemployed	13(52)	17(34)	

^**¶**^95% Confidence interval

**Table 3:** Comparing cholera exposure characteristics among cholera cases and controls using bivariate analysis, Addis Ababa, Ethiopia: 07 September to 01 October, 2017.

Exposures	Cases Number (%)	Controls Number (%)	COR at 95%CI^¶^
**Sharing drinking water source with at least one household**			
Yes	19(76)	28(56)	2.49(0.85–7.29)
No	6(24)	22(44)	
**Treating drinking water with chemicals**			
Yes	7(28)	23(46)	0.46(0.16–1.29)
No	18(72)	27(54)	
**Drinking holy water 5 days before onset of illness**			
Yes	12(48)	4(8)	10.62(2.93–38.50) *
No	13(52)	46(92)	
**Eating Raw vegetables 5 days before onset of illness**			
Yes	14(56)	10(20)	5.09(1.78–14.56)*
No	11(44)	40(80)	
**Eating Raw fruits 5 days before onset of illness**			
Yes	1(4)	9(18)	0.18(0.02–1.60)
No	24(96)	41(82)	
**Eating Raw meat 5 days before onset of illness**			
Yes	3(12)	8(16)	0.72(0.17–2.97)
No	22(88)	42(84)	
**Eating food outside home(restaurant, street vendor, work canteen) 5 days before onset of illness**			
Yes	12(48)	22(44)	1.17(0.45,3.08)
No	13(52)	28(56)	
**Sharing latrine with at least one household**			
Yes	18(72)	21(42)	3.55(1.26–10.03)
No	7(28)	29(58)	
**Washing hands with soap after visiting latrine**			
yes	17(68)	45(90)	0.23(0.07, 0.82)*
No	8(32)	5(10)	
**Contact history with cholera suspected person**			
yes	5(20)	1(2)	**12.25(1.34,111.57)**
No	20(80)	49(98)	
**Traveling history 5 days before onset of illness**			
Yes	5(20)	7(14)	1.53(0.43–5.43)
No	20(80)	43(86)	

^**¶**^95% Confidence interval

*statistically significant variables

Variables that were kept in multivariable model included consumption of raw vegetables and holy water, and washing hands with soaps. In our multivariable analysis, consumption of holy water (AOR: 20.5, 95%CI: **3.50,119.61**) and raw vegetables (AOR: 15.3, 95%CI: 3, 81.51) were identified as independent risk factors whereas washing hands with soap after visiting latrine (AOR: 0.04, 95%CI: 0.01, 0.25) was independent protective factor (Table 4).

**Table 4:** Comparing cholera exposure characteristics among cholera cases and controls using multivariate analysis, Addis Ababa, Ethiopia: 07 September to 01 October, 2017.

Exposures	Cases Number (%)	Controls Number (%)	AOR at 95%CI^¶^
**Drinking holy water 5 days before onset of illness**			
Yes	12(48)	4(8)	**20.5(3.50,119.61)**
No	13(52)	46(92)	
**Eating Raw vegetables 5 days before onset of illness**			
Yes	14(56)	10(20)	**15.3(3,81.51)**
No	11(44)	40(80)	
No	7(28)	29(58)	
**Washing hands with soap after visiting latrine**			
yes	17(68)	45(90)	**0.04(0.01,0.25)**
No	8(32)	5(10)	
No	20(80)	43(86)	

## Discussion

This cholera outbreak implicated cholera foodborne and waterborne transmission as both consumption of raw vegetables and contaminated holy water were associated statistically with the outbreak. Hand wash was found to be a protective factor against cholera demonstrating the role of hygiene in cholera control.

Fresh vegetables usually harbor natural non-pathogenic epiphytic microorganisms, but during growth, harvest, transport and further handling the produce can be contaminated with pathogens from animal and human sources [17]. Raw produces can be contaminated with *Vibrio cholerae* at any points of production chain, from farm to consumer’s mouth, with *Vibrio cholerae* being viable for 2–5 days on the contaminated produce [18]. In our investigation, we found that raw vegetables were independent risk factors for cholera outbreak; a finding consistent with results of earlier studies. Sinkala *et al*. [19] showed that raw vegetables were the primary vehicles of transmission for cholera during their cholera outbreak investigation in Zambia. In Nigeria, Dahiru and Sulaiman [20] demonstrated that 7% of the vegetable samples were positive for *V*.*cholerae*. Treating soil with organic fertilizers, irrigating vegetables with wastewater sources and, the ability of pathogens to persist and proliferate in vegetables can contaminate vegetables [21]. During our environmental investigation, we observed small vegetable farms in the compound of the patients and, they usually use organic fertilizers and irrigate with wastewater sources which might relate to contamination of the vegetables. Further investigation is needed to assess the level of bacterial contamination of vegetables in the study area to recommend plausible interventions.

Consist with our recent investigation finding that consumption of unsafe holy water was associated with the outbreak; previous studies have also shown that water source contamination is among the most common risk factors for cholera outbreak. Griffith *et al*., (6) demonstrated in their review of reported cholera outbreaks worldwide (1995–2005) that water source contamination, heavy rainfall and flooding, and population dislocation are the most common risk factors for cholera outbreak. In addition, study in Dhaka by Rafique *et al*. [22] demonstrated that infectious *V*. *cholerae* transmits within the household contacts of cholera-patients through drinking water as he confirmed through isolating *V*.*cholerae* from stored drinking water and water source samples. Furthermore, study in Ethiopia by Walle et al. [23] showed the association of cholera outbreak with unsafe drinking water.

Studies showed that *V*.*cholerae*, the causative agent of cholera, has been well established as the native flora of aquatic environments rendering drinking water to be risk factor for cholera outbreak [24]. Cholera spreads from these aquatic environments including water systems following heavy rain and flood causing cholera outbreak [6]. In our current outbreak investigation, we investigated holy water sources that were used by cholera cases and we observed that the water sources were flooded following heavy rains. In addition, we found that water specimens from these water sources yielded *V*.*cholerae* isolates, substantiating the fact that heavy rains and flood contributed to cholera spread causing its outbreak. We closed the holy waters to prevent further cholera infection and disinfected using chlorine solution to render them safe for use. This investigation suggests that holy water sources are potential sources of cholera outbreak needing continuous public health surveillance for new cases so as to detect and identify cases to contain the outbreak from spreading, especially during the seasons when cholera outbreak is suspected. Furthermore, this warrants the need to build flood embankments around holy water sources to prevent flooding and subsequent contamination of the water sources.

Infected individuals who are asymptomatic can contaminate foods during preparation but foods can also be contaminated by utensils, with *V*.*cholerae* being capable of persisting on utensils for 1–2 days [25]. Washing hands with soap is very crucial to interrupt the transmission of *V*.*cholerae* during food preparation, service and consumption. In our multivariate analysis of this cholera outbreak, washing hands with soap after visiting latrine was found to be an independent protective factor against cholera illness, a finding that is in agreement with previous studies. Chemeda *et al*. [8] demonstrated that washing hands with soap after visiting latrine reduced the risk of diarrhea by nearly 20% during his acute watery diarrhea outbreak investigation in Ethiopia. In addition, in Ethiopia, Beyene *et al*. [7] showed that washing hands with soap after visiting latrine reduced the risk of diarrhea by 85% during his acute watery diarrhea outbreak investigation. Hence, consistent and sufficient soap distribution and hand hygiene awareness creation campaigns would be significant interventions in cholera prevention and control strategies particularly during outbreak settings.

This outbreak investigation study was subject to three major limitations. Misclassification is one of the major biases that is a concern in case-control study, which occurs whenever subjects are erroneously categorized with respect to either exposure or disease status [26]. In our investigation, 18 case-patients were classified as cholera case-patients using case definition because stool samples were collected from only seven case-patients for laboratory culturing of *Vibrio cholerae* which may overestimate the attack rate as those with acute watery diarrhea caused by other than *Vibrio cholerae* could be classified as cholera case-patient. However, we assumed that this situation may not significantly distort the finding as the suspected cholera case-patients were recruited using standard cholera case definition for an area where there is cholera epidemic, supplemented with the apparent clinical signs of cholera. In addition, we found that controls were more likely to wash their hands with soap than cases, as washing hands with soap after visiting latrine was found to be a protective factor against cholera illness. This finding may suggest that the livelihood status of controls could be better than case-patients, enabling them to live in a more hygienic environment which reduces risk of exposures for cholera. We were unable to adjust the effect of economic status on the observed association because we did not collect income data for our study subjects. Nonetheless, we believed that the livelihood status of controls was not be able to confound the finding as the controls were recruited from the case-patients health center, implicating their similarity in income status. Furthermore, the finding of this study showed wider ranges in the strengths of association between the exposures and cholera illness (wider confidence intervals) which may be due to small sample size employed for our study needing precautions in interpretation, and investigation is needed using adequate sample size to have the best estimate in the target population.

## Conclusions

The finding of this study demonstrated cholera foodborne transmission via consumption of raw vegetables. In addition, this cholera outbreak was associated with waterborne exposures as suggested via consumption of contaminated holy water. Furthermore, the protective nature of hand hygiene against cholera as indicated by independent protective effect of washing hands after visiting latrine, a finding in our multivariate analysis, is an implication for transmission of cholera through foodborne exposures. We recommended washing or cooking vegetables before use and avoiding irrigating vegetables with sewages. In addition, we suggested boiling water before drinking. Moreover, We recommended educating the community on hygienic food handling. Furthermore, we recommended emphasizing hand washing with soaps after visiting latrine during community education campaigns particularly in outbreak situations. It was recommended building flood embankments around holy water sources to prevent flooding and subsequent contamination of the water sources.
